# Character strength traits, states, and emotional well‐being: A daily diary study

**DOI:** 10.1111/jopy.12933

**Published:** 2024-04-15

**Authors:** Lisa Wagner, Fabian Gander

**Affiliations:** ^1^ Department of Psychology University of Zurich Zurich Switzerland; ^2^ Jacobs Center for Productive Youth Development University of Zurich Zurich Switzerland; ^3^ Department of Psychology University of Basel Basel Switzerland

**Keywords:** character strengths, diary study, personality states, whole trait theory, within‐person variability

## Abstract

**Objective:**

Does whole trait theory work for character strengths? This study examines the daily within‐ and between‐person variability of the manifestations of positively valued lower‐order personality characteristics, namely character strengths, their convergence with trait character strengths, and their relationships to daily measures of affect.

**Background:**

Manifestations of personality traits vary both between‐ and within people. So far, research has focused on between‐person differences in character strengths, while within‐person differences have been neglected.

**Methods:**

German‐speaking participants (*N* = 199, 84.3% women; mean age = 26.0 years) participated in a two‐week daily diary study. They completed a baseline measure of character strength traits and daily measures of character strength states and positive and negative affect.

**Results:**

Results suggested that character strength traits converged well with aggregated states. Further, we observed high within‐person variability in most character strengths. The trait‐state convergence and the amount of within‐person variability were predicted by whether the character strengths were rather phasic (i.e., more dependent on situational characteristics) or rather tonic (i.e., less dependent on situational characteristics). Higher within‐person variability in character strengths was related to trait levels of perspective, honesty, social intelligence, and fairness. Regarding relationships between character strengths and affect, within‐person associations were widely parallel to previously reported between‐person associations and largely independent of trait levels of character strengths.

**Conclusion:**

These findings inform research on whole trait theory and character–strengths‐based interventions.

## 1 INTRODUCTION

In the present study, we use a whole trait theory perspective (Fleeson & Jayawickreme, 2015) to examine positively valued traits and states, namely character strengths. Many theoretical contributions and empirical studies suggested that character strengths traits represent the core characteristics of the “good life.” Also, the character strengths framework has been the basis of many practical applications (e.g., Ruch et al., 2020). At the same time, most of these studies relied on cross‐sectional examinations of character strengths and, thereby, on between‐person effects, while within‐person effects have been largely ignored. However, many relevant research questions—especially those related to practical applications—concern within‐person associations (e.g., Hamaker, 2012). For instance, do individuals experience an improvement in their well‐being when they exhibit a character strength more frequently than usual, such as behaving more gratefully, creatively, or humorously?

The current study examines the variability of character strength states within and between individuals, as well as their association with character strength traits. Character strength states refer to daily enactments of character strengths, and character strength traits refer to the enduring tendency to enact a character strength. This study also aims to enhance our understanding of the within‐person links between character strengths and emotional well‐being.

## 2 CHARACTER STRENGTH TRAITS AND STATES

Character strengths are a family of positively valued personality traits proposed by Peterson and Seligman (2004) in their Values in Action (VIA) classification. The 24 character strengths described in the VIA classification were derived in a collaborative effort to identify traits relevant to pursuing a “good life” that together represent good character. The character strengths are loosely organized under six broader virtues considered ubiquitous: (1) creativity, curiosity, judgment, love of learning, and perspective (organized under the virtue of wisdom and knowledge); (2) bravery, perseverance, honesty, and zest (virtue of courage); (3) love, kindness, and social intelligence (virtue of humanity); (4) teamwork, fairness, and leadership (virtue of justice); (5) forgiveness, humility, prudence, and self‐regulation (virtue of temperance); and (6) appreciation of beauty and excellence, gratitude, hope, humor, and spirituality (virtue of transcendence).

Character strengths show a substantial empirical overlap, yet no complete redundancy, with personality traits of the five‐factor model (McGrath et al., 2020; Ruch et al., 2023). In addition, character strengths share many characteristics with personality traits. For instance, the agreement between self‐reports and informant reports is highly comparable to what is typically found for personality traits of the five‐factor model (Buschor et al., 2013). However, character strengths also have some distinctive features. For example, their breadth is narrower—approximately at the level of five‐factor model facets– and they are explicitly evaluative traits, which are perceived as positively valued even in the absence of positive consequences (Stahlmann & Ruch, 2020).

### 2.1 Applying whole trait theory to character traits

Whole trait theory (Fleeson, 2001; Fleeson & Jayawickreme, 2015) posits that personality traits should be thought of as distributions of personality states, encompassing both an individual's mean and variability in enacting the personality trait. Personality states are characterized by sharing the same emotional, behavioral, and cognitive attributes as the corresponding traits, albeit with a briefer duration (Fleeson et al., 2015). In past studies, this briefer duration has been referring to behavior in the current moment or activity (e.g., Beckmann et al., 2021; Horstmann et al., 2021; Rauthmann et al., 2019), during the last few minutes or hours (e.g., Fleeson, 2001; Fleeson & Gallagher, 2009), during the past day (e.g., Howell et al., 2017; Judge et al., 2014; Nübold & Hülsheger, 2021; Ringwald et al., 2022), or the past week (e.g., Fleeson & Gallagher, 2009). The whole trait theory perspective allows accounting for consistency and variability in enacting personality traits. While the question of what is captured by aggregated states is still an ongoing debate, as demonstrated by Finnigan and Vazire (2018) and Breil et al. (2022), the idea that trait measures of the five‐factor model align closely with aggregated state measures of these traits is widely supported (e.g., Fleeson & Gallagher, 2009). Mean levels of traits can, thus, be understood as the location of distributions of personality states. While whole trait theory was developed to describe and explain broad personality traits, it has been demonstrated that the principles also apply to narrower constructs (e.g., creativity: Conner & Silvia, 2015; interpersonal trust: Fleeson & Leicht, 2006; or self‐criticism: Zuroff et al., 2016).

In the past, it has been questioned whether character strengths, such as honesty or fairness, can be conceptualized as stable and consistent traits. This skepticism arose from studies suggesting that even slight changes in situational factors might affect the extent to which individuals exhibit moral behavior (e.g., Latané & Rodin, 1969), as well as from research indicating low consistency in moral behavior (e.g., Hartshorne & May, 1928). As a result, the question arises as to whether whole trait theory (Fleeson, 2001; Fleeson & Jayawickreme, 2015), which was initially developed to describe and explain the personality traits of the five‐factor model, can also be extended to virtues, moral traits, or character strengths. Although it has been theoretically argued that whole trait theory should also apply to evaluative traits such as virtues or character strengths (Fleeson et al., 2015; Jayawickreme et al., 2014; Jayawickreme & Fleeson, 2017), there is little direct empirical evidence to support this claim so far. Exceptions were Meindl et al. (2015) and Prentice et al. (2020), who studied state expressions of moral behavior (i.e., honesty, fairness, compassion, and moral courage) and reported high consistency across time and situations; those who display relatively high moral behavior on one occasion tend to maintain their level of morality on other occasions. However, character strengths extend moral behaviors by also encompassing desirable (but not necessarily moral) behaviors, such as in the character strengths of creativity, love of learning, or zest. We do not know how character strengths states vary within and between persons, how their density distributions are best described, and how they relate to trait measures of character strengths. Therefore, this study aims to fill this gap and determine whether the assumptions of whole trait theory also apply to character strengths.^1^

### 2.2 Character strength traits as aggregates of character strength states

To date, research on character strengths has primarily focused on studying between‐person effects. Bleidorn and Denissen (2015) were the first to study character traits at the state level. However, they did not assess them in terms of character strength states but only at the more abstract level of virtues. While their results support the idea that morally valued traits can be described using density distributions and that virtue states vary across different social roles, they do not address the convergence of aggregated states with trait measures. In a study focused on the educational context, Wagner and Ruch (2023) investigated character strength states across five days in secondary school students. They found a medium‐sized average convergence between daily enactments of character strengths in the context of school (assessed at the end of each school day) and a global trait measure of character strengths. This finding provides initial support for the idea that character strength traits can be described as aggregated character strength states. However, its contribution to this question is limited by the assessment of states in the context of school.

### 2.3 Differences among character strengths in within‐ and between‐person variability and the emergence of character strengths

Although this question has not yet been addressed empirically, it can be assumed that there is variation among character strengths regarding the amount of between‐ and within‐person variability in states. Peterson and Seligman (2004) suggest a distinction in the emergence of character strengths between *tonic* and *phasic* strengths, which highlights the relevance of studying character strength states and their variability (“We must be interested not only in variation across people but also within people;” Park & Peterson, 2007, p. 295). *Tonic* character strengths can be enacted on many occasions and do not require specific situational cues or demands. In contrast, *phasic* strengths are enacted in response to specific situational cues or demands. For instance, most situations can be approached zestfully, whereas forgiveness can only be enacted in specific situations (e.g., when some form of transgression is perceived). Peterson and Seligman (2004) suggested zest, kindness, curiosity, modesty, and humor as examples of tonic strengths, and bravery, teamwork, judgment, and forgiveness as examples of phasic strengths.

To test these assumptions, Arbenz et al. (2023) asked experts to rate the emergence of character strengths, that is, the degree to which a character strength is tonic or phasic. Forgiveness, bravery, teamwork, and leadership were perceived as most phasic, and kindness, modesty, honesty, hope, curiosity, spirituality, social intelligence, and perspective were perceived as most tonic. Although it is implied that phasic strengths would vary more strongly within an individual, it has not been investigated yet whether tonic and phasic character strengths differ in their observed within‐person variability or whether tonic traits show a stronger relationship between traits and aggregated states.

### 2.4 Associations of character strength traits with variability in states

Intraindividual personality variability, that is, the extent to which an individual varies in their enactment of personality states, has been described as a trait on its own (Baird et al., 2006; Fleeson, 2001; Noftle & Fleeson, 2015). This variability in personality states has been proposed to underlie the ability to understand others' mental states (Wundrack et al., 2018). Wundrack et al. (2018) suggested in a theoretical account that higher variability in personality states means that such individuals “experience more diverse thoughts, feelings, and behaviors” (p. 7), which is assumed to be conducive to perspective‐taking in two ways. First, it is assumed to reduce egocentric bias (indicating a stronger readiness to move away from one's perspective when considering another's perspective). Second, a larger variability in personality states is suggested to facilitate perspective‐pooling as it leads to a stronger familiarity with various perspectives. These theoretical assumptions, however, have yet to be tested empirically. Given that some character strengths refer to perspective‐taking, the present study investigated whether character strengths traits are related to intraindividual variability in character strengths states.

## 3 CHARACTER STRENGTHS AND EMOTIONAL WELL‐BEING

Most studies on the associations between character strengths and subjective well‐being have focused on the cognitive component of subjective well‐being (e.g., Baumann et al., 2020; Bruna et al., 2019; Park et al., 2004; Proyer et al., 2011). These measures usually ask for a trait‐like overall evaluation of life satisfaction. However, when studying correlates of character strengths states, emotional well‐being (such as positive and negative affect), which can also be measured meaningfully at shorter intervals, seems more important. Several studies have provided evidence for robust associations of character strengths with trait‐like indicators of emotional well‐being (Azañedo et al., 2014; Khumalo et al., 2008; Littman‐Ovadia & Lavy, 2012; Martínez‐Martí & Ruch, 2017). To provide an overview of the general associations across studies, we computed meta‐analytic averages of the correlations between character strengths traits (as assessed by the VIA‐IS; Peterson & Seligman, 2004) and trait‐like positive and negative affect (Table 1).

**TABLE 1:** Meta‐analytic averages of the correlations of character strengths with emotional well‐being in previous studies and 95% confidence intervals.

	Meta‐analytic *r* with trait‐like positive affect (PA)	Meta‐analytic *r* with trait‐like negative affect (NA)
Creativity	0.41 [0.34, 0.48]	−0.07 [−0.09, −0.04]
Curiosity	0.51 [0.41, 0.61]	−0.22 [−0.37, −0.08]
Judgment	0.34 [0.22, 0.45]	−0.13 [−0.21, −0.06]
Learning	0.39 [0.31, 0.47]	−0.12 [−0.28, 0.04]
Perspective	0.44 [0.41, 0.46]	−0.19 [−0.21, −0.16]
Bravery	0.40 [0.35, 0.44]	−0.18 [−0.34, −0.01]
Perseverance	0.40 [0.38, 0.42]	−0.21 [−0.41, 0.00]
Honesty	0.29 [0.19, 0.39]	−0.19 [−0.29, −0.10]
Zest	0.59 [0.45, 0.72]	−0.26 [−0.47, −0.04]
Love	0.36 [0.23, 0.50]	−0.17 [−0.32, −0.02]
Kindness	0.30 [0.25, 0.35]	−0.14 [−0.25, −0.04]
Social int.	0.42 [0.33, 0.51]	−0.16 [−0.30, −0.03]
Teamwork	0.28 [0.19, 0.36]	−0.19 [−0.33, −0.05]
Fairness	0.21 [0.11, 0.32]	−0.14 [−0.21, −0.07]
Leadership	0.34 [0.31, 0.38]	−0.14 [−0.15, −0.12]
Forgiveness	0.21 [0.12, 0.31]	−0.20 [−0.39, −0.01]
Humility	0.07 [−0.10, 0.25]	−0.10 [−0.20, 0.00]
Prudence	0.18 [0.01, 0.36]	−0.15 [−0.33, 0.02]
Self‐reg.	0.35 [0.29, 0.40]	−0.22 [−0.48, 0.04]
ABE	0.32 [0.24, 0.40]	−0.05 [−0.16, 0.07]
Gratitude	0.40 [0.38, 0.42]	−0.22 [−0.33, 0.02]
Hope	0.53 [0.38, 0.69]	−0.35 [−0.59, −0.11]
Humor	0.39 [0.30, 0.49]	−0.20 [−0.35, −0.05]
Spirituality	0.20 [0.06, 0.34]	−0.11 [−0.28, 0.05]

*Note*: *N* for correlations with PA = 1860. *N* for correlations with NA = 1497. Studies included for estimating the correlations with PA (*k* = 4): Azañedo et al. (2014), Khumalo et al. (2008), Littman‐Ovadia and Lavy (2012), and Martínez‐Martí and Ruch (2017). Studies included for estimating the correlations with NA (*k* = 3): Azañedo et al. (2014), Khumalo et al. (2008), and Littman‐Ovadia and Lavy (2012). Khumalo et al. (2008) used the 20‐item Affectometer (Kammann & Flett, 1983) to assess PA and NA, and the remaining studies used the 20‐item PANAS (Watson et al., 1988). Meta‐analytic averages were computed using the psychmeta package (Dahlke & Wiernik, 2019). Individual study results are given in the online supplementary in Table S1.

Abbreviations: ABE, appreciation of beauty and excellence; Self‐reg., self‐regulation; Social int., social intelligence.

As Table 1 shows, the point estimates of the meta‐analytic average correlations of character strengths with positive affect (PA) were exclusively positive and substantially higher than the (negative) correlations with negative affect (NA). Zest, hope, curiosity, perspective, social intelligence, creativity, gratitude, perseverance, and bravery showed the strongest average correlations with positive affect (*r*s ≥ 0.40). Hope, zest, self‐regulation, curiosity, gratitude, perseverance, forgiveness, and humor showed the strongest average correlations with negative affect (*r*s ≤ −0.20).

### 3.1 Character strengths states and emotional well‐being

All previous studies examining the relationship between character strengths and emotional well‐being have utilized cross‐sectional designs and primarily focused on between‐person variation. This perspective only allows investigating whether those with higher levels of hope, for example, experience fewer negative emotions in general. However, it does not allow addressing whether being more hopeful than usual on a particular day is also related to experiencing fewer negative emotions compared to one's average level. Studying these relationships from a state perspective enables us to distinguish variance at the between‐ and within‐person levels. From a practical standpoint, within‐person effects are especially significant. For instance, a gratitude‐based intervention is predicated on the assumption that an individual will experience higher levels of well‐being if they exhibit acts of gratitude more frequently.

Moreover, asking about state (rather than trait) levels does not require respondents to aggregate ratings over extended periods, which can reduce susceptibility to memory biases (Bolger et al., 2003). Research has also shown that relationships between constructs found at the between‐person level are not necessarily parallel to findings obtained at the within‐person level (Curran & Bauer, 2011; Hamaker, 2012). The first evidence for within‐person associations of character‐related states with positive and negative affect was found in Bleidorn and Denissen's (2015) study on virtue states, which analyzed these associations at the abstract level of virtues. However, character strengths assigned to one virtue substantially differ in their association with emotional well‐being (e.g., as shown in Table 1, hope shows one of the strongest and spirituality one of the weakest relationships, yet both are assigned to the virtue of transcendence in the VIA classification). We, therefore, build on this work to examine whether the associations between character strengths and emotional well‐being from an interpersonal perspective also apply to intrapersonal associations.

### 3.2 Differential relationships between character strength states and emotional well‐being

When examining the associations between personality states and well‐being, one important question is whether a person's trait level moderates these relationships. For instance, is enacting gratitude associated with higher levels of positive affect for everyone or only for those with high levels of trait gratitude? Several theoretical perspectives on this question have suggested the relevance of an individual's trait level. First, the behavioral concordance model (Moskowitz & Coté, 1995) argues that individuals experience more positive affect when they enact personality states congruent with their trait levels; for instance, a person low on extraversion should enjoy acting introvertedly. A second theory, the contra‐trait effort hypothesis (Gallagher et al., 2011), assumes that enacting personality states that differ from an individual's trait levels is more effortful than enacting trait‐congruent personality states. For example, according to this hypothesis, an individual low on extraversion should feel tired after behaving in an extraverted manner.

Empirical studies have yielded mixed results. Some studies found evidence in line with moderation effects of trait levels. For instance, Jacques‐Hamilton et al. (2019) reported that introverted individuals showed weaker increases in positive affect following an intervention that asked them to enact extraversion more frequently in their daily lives. However, most studies that either experimentally manipulated extraversion states or used experience sampling have concluded that the relationships between personality states and affect were mostly independent of trait levels (e.g., Epley & Schroeder, 2014; Fleeson et al., 2002; Howell et al., 2017; Margolis & Lyubomirsky, 2020; van Allen et al., 2021). Studies that included traits outside of extraversion have also not found evidence for moderating effects of traits (e.g., Kritzler & Luhmann, 2021).

In the present study, we aim to test this question with respect to character strengths. As noted by previous studies (e.g., Jacques‐Hamilton et al., 2019; Margolis & Lyubomirsky, 2020), knowing whether individuals differ in their relationships between personality state and affect based on their trait levels is crucial for designing effective interventions based on prompting the enactment of personality states. This question is particularly relevant for character strengths, given that strengths‐based interventions were found to increase well‐being (for a review, see Schutte & Malouff, 2019), but the question of which character strengths should be targeted—those in which the individual has high levels or others—is still debated (Ruch et al., 2020). Knowing whether the correlates of enacting character strengths depend on an individual's character strengths trait levels would help inform this question.

## 4 THE PRESENT STUDY

Jayawickreme and Fleeson (2017) asked, “Does whole trait theory work for the virtues?” (p. 75) and this study aims to address this question empirically by examining the daily within‐ and between‐person variability of character strengths and their associations with trait character strengths. Building on Jayawickreme and Fleeson (2017), we aim to test whether the notion that average personality state scores represent stable, individual differences in personality traits (Fleeson, 2001; Fleeson & Gallagher, 2009; Jayawickreme et al., 2019; Rauthmann et al., 2019) also generalizes to narrower, positively valued traits; that is, character strengths.

- *Research question 1*: Can character strength traits be described as aggregates of character strength states?
- *Research question 2*: (i) Do character strengths states differ regarding the amount of within‐person variability? And does the emergence of character strengths (i.e., whether a strength is rather tonic or phasic) go along with (ii) higher variability in character strengths states, and (iii) does emergence moderate the association between traits and states?

While research question 2 (i) examines differences among character strengths in their variability, (ii) examines to what degree these differences in variability could be explained by emergence. Further, (iii) examines whether emergence could also explain differences among the associations between states and traits that were studied in research question 1.

- *Research question 3*: How does variability in character strength states relate to character strength traits?

Research question 3 extends the previous research questions by examining the role of each character strength trait for state variability, that is, for example, whether trait creativity goes along with higher variability in character strengths states.

Further, this study extends research on the relationships between character strengths and emotional well‐being by studying within‐person relationships between character strengths states and affective states (Research questions 4 and 5).

- *Research question 4*: Can the between‐person relationships of character strengths with emotional well‐being (i.e., positive and negative affect) be replicated at the within‐person level?
- *Research question 5*: Does the trait level of a character strength moderate the relationships between character strength states and emotional well‐being?

We did not preregister any hypotheses and, therefore, consider our analyses on these research questions exploratory.

## 5 METHOD

We report how we determined our sample size, all data exclusions, all manipulations, and all measures in the study. The R code for the main analyses and a codebook are available on the Open Science Framework (https://osf.io/mdtac/). The raw data, unfortunately, cannot be made publicly available because the consent form used in this study explicitly excluded the disclosure of data to third parties.

### 5.1 Participants

We initially aimed to collect at least 100 participants (based on an a priori power analysis for detecting between‐person correlations of *r* = 0.30 with a power of ≥0.80) to account for dropouts and missing data. We were more successful than anticipated in recruiting participants: A total of 199 participants (84.3% women) aged 18 to 67 (*M* = 26.02, *SD* = 10.43) provided data. They were predominantly Swiss (76.3%) or German (16.2%) citizens; 7.6% had another nationality. Regarding their highest education, 17.6% held a degree from a university or a university of applied sciences, 76.8% held a diploma allowing them to attend a university or a university of applied sciences, 3.5% completed vocational training, and 2.0% completed secondary school. Most participants were students (75.2%) or employed (19.2%), while the remaining participants were homemakers, interns, unemployed, in vocational training, on sick leave, retired, or did not answer this question.

The 199 participants completed a total of 2386 daily measures (out of 2786 possible days), with an average of *M* = 12.00 daily measures (*SD* = 2.88). Sensitivity analyses suggested that, on the between‐subject level, we could detect effect sizes of *r* ≥ 0.20 with a power of at least 0.80. For cross‐level interactions in multilevel models, we conducted power simulations (Green & MacLeod, 2016), which suggested that we could detect standardized interaction effects of *β* ≥ 0.07 with a power of at least 0.80.

### 5.2 Instruments

The *VIA Inventory of Strengths* (VIA‐IS; Peterson & Seligman, 2004; German version by Ruch et al., 2010) assesses the 24 character strengths described in the VIA classification as traits with 10 items per strength. It uses a 5‐point Likert‐style scale ranging between 5 = “very much like me” and 1 = “very much unlike me.” A sample item is “I find the world a very interesting place” (curiosity). Ruch et al. (2010) reported a good convergence between self‐rating and informant ratings and showed that self‐ratings are not strongly influenced by social desirability. While there is some discussion regarding the higher‐order factorial structure of the VIA‐IS (e.g., McGrath, 2014), the present study focuses on the lower‐level character strengths traits. Internal consistencies in the present study ranged between *ω* = 0.74 and *ω* = 0.95 (median *ω* = 0.81).

The *Character Strengths State Rating Form* (CSSRF) assesses the 24 character strengths from the VIA classification as daily states using one item per strength. We opted to focus on character strengths‐related behaviors in daily accounts rather than shorter time frames (e.g., the last hour), as displaying some strengths may necessitate specific, relatively uncommon situations. For instance, forgiveness typically involves situations where one has been wronged, while leadership requires interactions with other people who can be led. The CSSRF was developed based on the Character Strengths Rating Form (CSRF; Ruch et al., 2014), an established measure assessing trait levels of the 24 character strengths using one item per strength. The CSRF has shown good convergence with a 240‐item measure of character strengths (Ruch et al., 2014), similar self‐informant convergence compared to the 240‐item measure (Wagner et al., 2020) as well as high test–retest stability across two years (Gander et al., 2020; median *r*_tt_ = 0.46, ranging from 0.37 to 0.69). Given the good psychometric properties of the CSRF on which the CSSRF is based on, and the fact that several earlier studies also used 1‐item measures for narrow traits (e.g., creativity: Conner & Silvia, 2015) and broader traits (e.g., HEXACO‐dimensions: Sherman et al., 2015), we were confident that the CSSRF serves as an acceptable measure of strength states. Each CSSRF item uses the prefix “Today I have shown …” followed by the item from the CSRF, which consists of a detailed description of the respective character strengths containing multiple behaviors representing manifestations of the character strength. A sample item for the character strength of creativity is “Today I have shown creativity (originality, ingenuity; Creative people have a distinctive way of thinking about new problem‐solving paths and often have creative and original ideas. They are not satisfied with conventions).” The CSSRF uses a 7‐point Likert‐style scale with the following response options: 1 = “not at all,” 2 = “very rarely,” 3 = “rarely,” 4 = “occasionally,” 5 = “frequently,” 6 = “very frequently,” 7 = “all the time.”

*Positive and Negative Affect* (PA/NA) was assessed using a measure by Mroczek and Kolarz (1998). It consists of six items (three assessing PA and three assessing NA), each asking for the frequency of experiencing positive and negative states during the day. All items were answered on a 4‐point scale that ranges between 1 = “none of the time” and 4 = “all of the time.” A sample item is “How much of the time did you feel…nervous today?” (NA). Internal consistencies ranged between *ω* = 0.88 and 0.94 (median across days *ω* = 0.90) for positive affect and between *ω* = 0.77 and 0.91 (median across days *ω* = 0.86) for negative affect.

### 5.3 Procedure

According to the local ethics committee guidelines, the present study did not require ethical review. Participants provided informed consent, and participation was voluntary. They could receive partial course credit and individual feedback on their character strengths as an incentive for participation. We recruited participants via social media, mailing lists, and postings in public places. After completing a baseline assessment (demographic variables, character strength traits), we invited participants via e‐mail to complete a daily questionnaire over 14 consecutive days (character strength states, positive and negative affect). This daily questionnaire could be completed between 4 p.m. and 2 a.m. the following day.

The data presented here were collected as part of a larger study in February and March of 2018 and partially overlap with those presented in Gander et al. (2022), which also used the CSSRF data. However, there is no overlap between the research questions of the two manuscripts. Both the baseline assessment and the daily questionnaires contained additional measures not relevant to this study, namely single‐item measures of character strengths functions (see Gander et al., 2022) in the diary part and everyday indicators of virtues (e.g., number of books one possesses as an indicator for the virtue of wisdom and knowledge) and a measure of life satisfaction (Diener et al., 1985) at the baseline assessment.

### 5.4 Data analysis

For all analyses, we do not report p‐values but instead report 95% confidence intervals (or credible intervals for Bayesian models, respectively) for estimated parameters. In all analyses using Level‐1 (i.e., non‐aggregated) data, we controlled for temporal effects by including the fixed effects of time (i.e., the number of days since the study started) to rule out the possibility that repeated testing or other longitudinal changes might have affected the results (e.g., Curran & Bauer, 2011; Denissen et al., 2019).

For research question 1, we computed zero‐order correlations of aggregated data to examine the associations between character strengths states and traits. We used the guidelines by Gignac and Szodorai (2016) for interpreting correlations; that is, *r* ≥ 0.10 denotes a small effect, while *r* ≥ 0.20 a medium, and *r* ≥ 0.30 a large effect. For research question 1, we used correlations *r* ≥ 0.30 as a cutoff: Fleeson and Gallagher (2009) reported meta‐analytic correlations between 0.42 and 0.56 for the associations between the five‐factor model traits and states. Since we used 1‐item state measures that are expected to be less reliable, we decided to use a correlation of *r* ≥ 0.30 as a threshold to determine whether the associations between the states and the traits were consistent with the assumption of whole trait theory that traits represent aggregates of states.

We computed Bayesian censored location‐scale models to analyze research questions 2 and 3 (Williams et al., 2020). Location‐scale models allow simultaneous estimation of the mean and variability of a dependent variable. One major advantage of these models is that the correlation between a person's mean and their variability is considered in the model estimate, which helps overcome the problem that standard deviations are often strongly dependent on the mean (Baird et al., 2006). The choice of censored models, as recommended by Mader et al. (2023), was driven by the nature of our empirical data, which is inherently bounded by the response scale. This often leads to ceiling or floor effects, where a substantial number of responses cluster at the maximum or minimum of the scale, respectively. In censored models, scores at these extremes are treated as indicators of “true” values that exist at, or potentially beyond, these scale boundaries. This approach allows us to represent the underlying distribution of responses more accurately, acknowledging that the observed variances in our data are constrained by the scale limits rather than being purely a function of the participants' responses. This detailed consideration of scale boundaries in our statistical modeling addresses potential misrepresentations of variability and mean estimates that could arise in standard uncensored analyses. For all Bayesian models, we used the default priors (flat or weakly informative) and ran the model with four chains using a total of 2000 iterations, of which 1000 were used for warm‐up. In cases where the chains did not converge well (potential scale reduction factor *R̂* ≥ 1.05), we ran the models again using 3000 iterations. All reported models resulted in good convergence (all *R̂* < 1.05 for the coefficients of interest)^2^ and precision (effective sample size [ESS] > 500 for most models, for estimations of variability of individual strengths: ESS > 70).

To address the first part of research question 2, we separately predicted the mean and variability of states by the intercept and measurement time point for each character strength state. We compared the resulting credibility intervals to examine whether some character strengths showed more within‐person variability than on average (i.e., than the average character strength).

For the second and third part of research question 2 (the role of emergence in predicting variability and moderating the association between states and traits), we predicted the level and variability of states by the *z*‐standardized trait level (across all character strengths), the *z*‐standardized emergence of character strengths (average expert ratings for emergence for each strength were taken from Arbenz et al., 2023, Table 1), and the interaction between the trait level and emergence. In these analyses, we allowed for random intercepts for each person and each character strength, thus considering character strengths as random factors.^3^

To analyze whether character strengths traits relate to the variability in character strengths states (research question 3), we again computed a series of location‐scale models predicting the level and variation of character strengths states (across all character strengths) separately by each character strength trait. These models allowed for random intercepts for each person.

To examine the associations of affect with character strengths states (research question 4), we predicted positive or negative affect by between‐ and within‐person parts of character strengths states in multilevel models. We separated character strengths state levels into parts that vary between person (i.e., a person's average level across all days) and the parts that vary within persons (i.e., a person's day‐to‐day deviation from their average). Within‐person parts were centered on the person mean, and between‐person parts on the grand mean (Curran & Bauer, 2011). The multilevel models allowed for a random intercept for each person and random slopes for the within‐person part of states (Heisig & Schaeffer, 2019).

To examine whether character strengths traits moderate the association between states and well‐being (research question 5), we conducted similar analyses as for research question 4: We predicted positive and negative affect by character strengths traits, within‐person parts of states, and their interaction while allowing for random intercepts for the person and random slopes for the within‐person parts of states.

We used R (Version 4.3.0; R Core Team, 2023) and the R‐packages *brms* (Version 2.19.0; Bürkner, 2021), *correlation* (Makowski et al., 2020), *DescTools* (Version 0.99.48; Signorell, 2023), *ggforestplot* (Version 0.1.0; Scheinin et al., 2023), interactions (Version 1.1.0; Long, 2021), *lme4* (Version 1.1.33; Bates et al., 2015), *lubridate* (Version 1.9.2; Grolemund & Wickham, 2011), *MBESS* (Version 4.9.2; Kelley, 2022), *papaja* (Version 0.1.1; Aust & Barth, 2022), *parameters* (Version 0.21.0; Lüdecke et al., 2020), *psych* (Version 2.3.3; Revelle, 2023), *psychmeta* (Version 2.6.5; Dahlke & Wiernik, 2019); *simr* (Version 1.0.7; Green & MacLeod, 2016), *sjPlot* (Version 2.8.14; Lüdecke, 2023), and *tidyverse* (Version 2.0.0; Wickham et al., 2019) for our analyses.

## 6 RESULTS

### 6.1 Relationships between states and traits of character strengths (research question 1)

**FIGURE 1:**
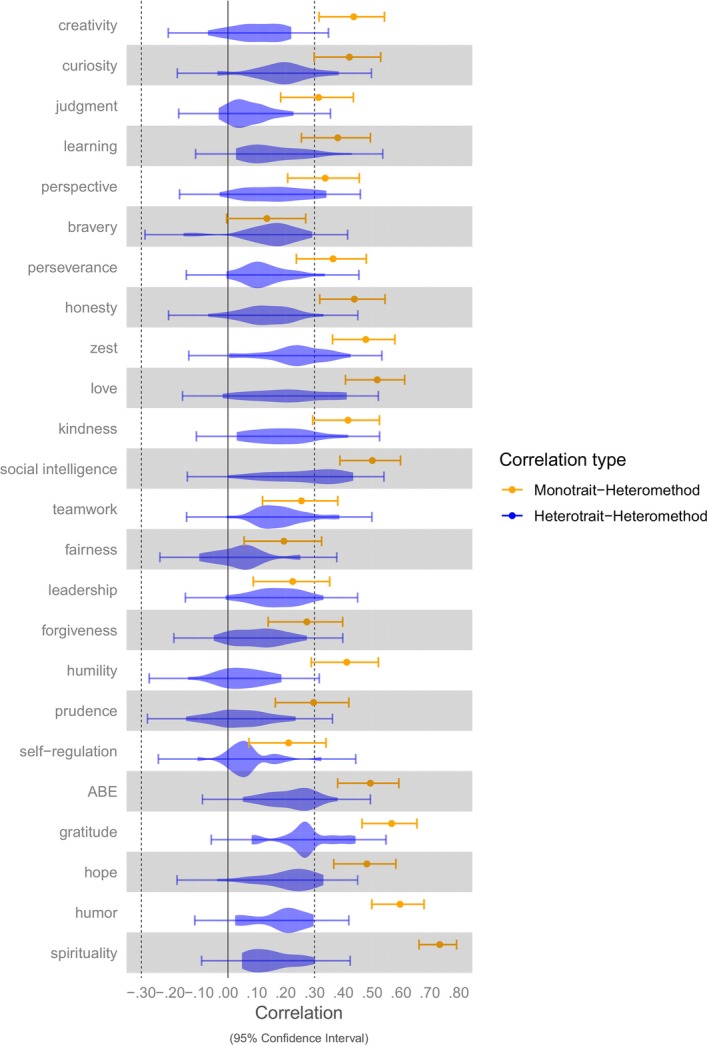
Correlations of Traits with States of the Same and the Remaining Character Strengths. *N* = 199 participants. The zero‐order correlations between a trait and the aggregated states of the same strength (monotrait‐heteromethod) and associated 95% confidence intervals are given in orange (e.g., first row: the correlation of trait creativity with aggregated state creativity). The correlations of a trait with aggregated states of different strengths (i.e., heterotrait‐heteromethod relationships) with associated 95% confidence intervals are given in blue (e.g., first row: the correlations of trait creativity with aggregated states of all other 23 character strengths). ABE, appreciation of beauty and excellence; Learning, love of learning.

Correlations of traits with aggregated states of the same character strengths (“monotrait‐heteromethod”) and the remaining character strengths states (“heterotrait‐heteromethod”) are given in Figure 1. Correlations among character strengths states and character strengths traits (“heterotrait‐monomethod”) are given in the online supplementary Table S5.

Figure 1 shows that for all strengths, the association between traits and aggregated states of the same strength was at least of a large effect size (*r* ≥ 0.30) for 18 out of the 24 strengths, suggesting a good fit with the assumption of whole trait theory based on the predefined cutoff criterion. Exceptions were bravery, teamwork, fairness, leadership, forgiveness, and self‐regulation, which yielded positive but smaller associations. For 15 strengths, the daily display of the strength also showed the numerically strongest relationship to the trait score of the same strength.

### 6.2 The variability of character strengths (research question 2)

**FIGURE 2:**
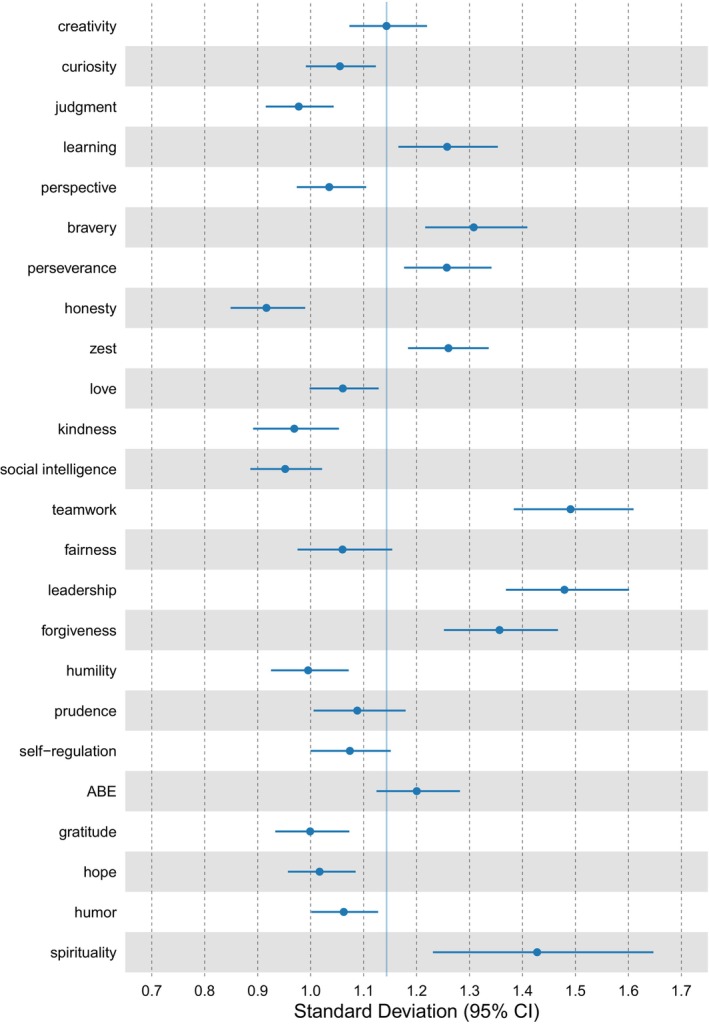
Variability of character strengths states and traits. *N* = 199 participants (2386 data points for daily measures). Given are within‐person standard deviations and 95% credibility intervals. ABE, appreciation of beauty and excellence; Learning, love of learning. The colored vertical line denotes the average standard deviation across all character strengths.

The within‐person variation of daily ratings based on location‐scale models is given in Figure 2 (see online supplementary Table S2 for means, standard deviations, and intra‐class‐correlations).

Figure 2 shows that for most character strengths, the 95% credibility interval of the within‐person standard deviation did not overlap with the average within‐person standard deviation across all character strengths, suggesting differences among character strengths regarding their variability. For example, the character strengths of spirituality, leadership, and teamwork varied more strongly within persons than the average across all character strengths. In contrast, other character strengths, such as for example, honesty, social intelligence, and kindness varied less within persons. Thus, people experience relatively large differences in their daily display of, for instance, leadership and teamwork, while only smaller fluctuations in honesty and social intelligence are experienced.

Associations of levels and variability of character strengths states with trait levels and estimations of emergence (ratings for emergence were taken from Arbenz et al., 2023; see Table S1) are given in Table 2.

**TABLE 2:** Predicting levels and variability of character strengths by traits levels and emergence.

	*β*
Location fixed effects	
Intercept	−0.04 [−0.30, 0.19]
Time	0.01 [0.01, 0.01]
Trait level	0.20 [0.19, 0.21]
Emergence	0.11 [−0.14, 0.35]
Trait level × Emergence	0.02 [0.01, 0.02]
Scale fixed effects	
Intercept	−0.16 [−0.25, −0.07]
Time	−0.01 [−0.01, −0.01]
Trait level	0.00 [−0.01, 0.01]
Emergence	−0.08 [−0.15, −0.01]
Trait level × Emergence	0.01 [0.01, 0.02]
Correlation (intercept & variability)	−0.41 [−0.51, −0.28]

*Note*: Given are standardized fixed effects from location‐scale models and 95% credible intervals. Emergence = Low scores denote a rather phasic trait (more variable); high scores denote a rather tonic (less variable) trait (ratings for emergence were taken from Arbenz et al., 2023).

Table 2 shows that the level of character strengths states (location effects) was predicted by trait levels (see research question 1) and the interaction between trait levels and emergence, suggesting that the association between traits and states was stronger for strengths that are more tonic (i.e., less variable) as compared to strengths that are more phasic (i.e., more variable). The variability of character strengths states (scale effects) was mostly predicted by the emergence of a character strength, suggesting that for more phasic strengths, more variability in states is reported than for more tonic strengths. Additionally, there was a small interaction of emergence with trait levels. Finally, intercept and variability of states were negatively correlated, suggesting that higher overall levels in states go along with less variability.

### 6.3 Relationships between character strength traits and variability in strength states (research question 3)

Associations of character strengths traits with variability in character strengths states are given in Table 3 (see online supplementary Table S3 for full results).

**TABLE 3:** Relationships between character strength traits with variability in character strength states.

	*β*
Creativity	0.01 [−0.04, 0.07]
Curiosity	0.02 [−0.03, 0.07]
Judgment	0.03 [−0.02, 0.08]
Learning	0.04 [−0.01, 0.08]
Perspective	0.08 [0.03, 0.12]
Bravery	0.04 [−0.01, 0.09]
Perseverance	0.04 [−0.01, 0.08]
Honesty	0.08 [0.03, 0.12]
Zest	0.00 [−0.05, 0.05]
Love	0.00 [−0.05, 0.05]
Kindness	0.02 [−0.03, 0.08]
Social intelligence	0.06 [0.01, 0.11]
Teamwork	0.01 [−0.03, 0.06]
Fairness	0.05 [0.01, 0.10]
Leadership	0.04 [−0.01, 0.10]
Forgiveness	−0.01 [−0.05, 0.04]
Humility	0.00 [−0.04, 0.05]
Prudence	0.01 [−0.04, 0.06]
Self‐regulation	0.03 [−0.02, 0.09]
ABE	0.00 [−0.05, 0.04]
Gratitude	−0.01 [−0.06, 0.04]
Hope	0.00 [−0.05, 0.05]
Humor	0.02 [−0.03, 0.07]
Spirituality	−0.01 [−0.06, 0.04]

*Note*: *N* = 199 participants (2386 data points for daily measures). Given are standardized fixed effects (and 95% credible intervals) from location‐scale models predicting the variability of states (i.e., scale effects) across all character strengths by traits while allowing for random intercepts for the person.

Abbreviations: ABE, appreciation of beauty and excellence; Learning, love of learning.

Table 3 shows that those who reported higher trait levels of perspective, honesty, social intelligence, and fairness also reported higher overall variability in character strengths states.

### 6.4 Relationships between character strength states and affect (research question 4)

**FIGURE 3:**
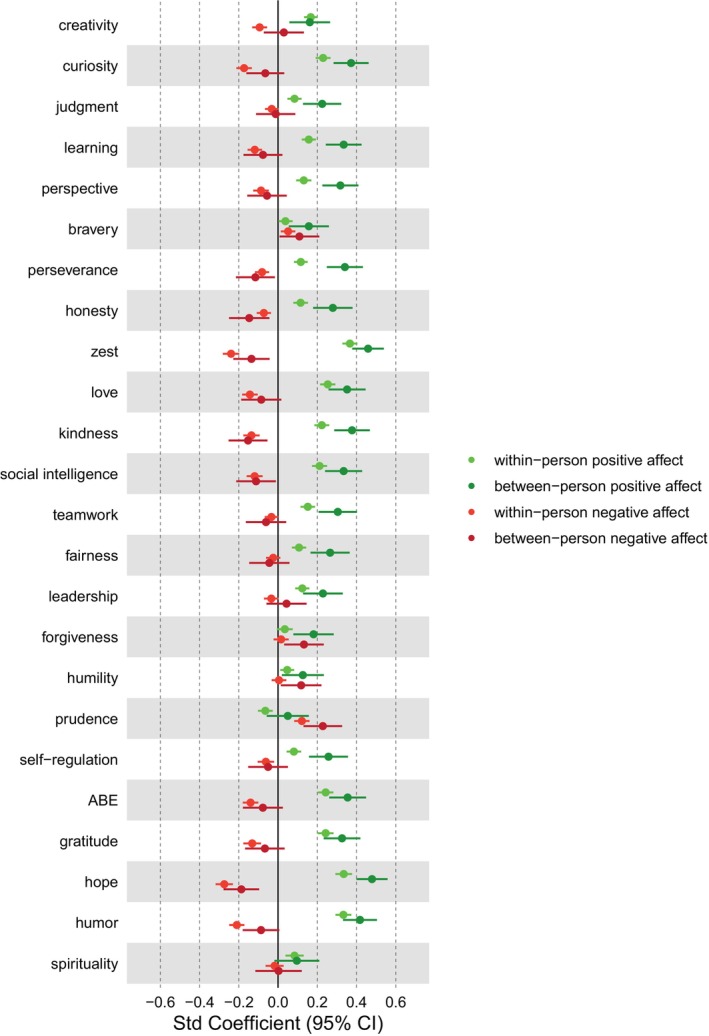
Relationships of between‐ and within‐person parts of character strength states with positive and negative affect. *N* = 199 participants (2386 data points for daily measures). Given are standardized fixed effects from multilevel models and 95% confidence intervals. ABE, appreciation of beauty and excellence; Learning, love of learning.

Associations of between‐ and within‐person parts of character strengths states with state positive and negative affect are given in Figure 3.

Figure 3 shows that the within‐person parts of most character strengths were positively related to positive affect. The exceptions were bravery and forgiveness, for which no effect was found, and prudence, for which a negative relationship was observed. The most pronounced positive effects were reported for zest, hope, humor, curiosity, love, gratitude, kindness, social intelligence, and appreciation of beauty and excellence. This finding means that, for example, on days when an individual is more curious than usual (i.e., higher than on their individual average), they experience more positive affect. While the between‐person associations of character strengths were overall higher than the within‐person associations, they showed a similar pattern: Except for prudence and spirituality, all character strengths positively related to positive affect on the between‐person level. Thus, for instance, people who reported higher average levels of curiosity than others also reported higher average levels of positive affect.

Within‐person parts of most character strengths also negatively related to negative affect, although the associations were smaller overall. The strongest negative effects were reported for zest, hope, humor, and curiosity, while prudence went along with more negative affect. Thus, prudent people report higher negative affect, and people also experience more negative affect on days when they are more prudent than usual. Again, between‐person effects were largely parallel to within‐person effects, and overall, associations of character strengths with negative affect mirrored associations with positive affect with the opposite sign.

### 6.5 Moderation effects of character strength traits on the relationship between character strength states and affect (research question 5)

**FIGURE 4:**
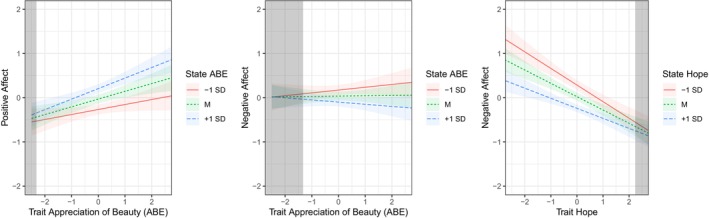
Interaction effects between character strength traits and states on affect. *N* = 199 participants (2386 data points for daily measures). Given are simple slopes (standardized effects) from multilevel models and 95% confidence intervals. For all trait levels *outside* the shaded areas, the association between the state and the dependent variable is significant. ABE, appreciation of beauty and excellence.

Results suggested few moderation effects (see Figure 4 for exemplary plots and Table S4 for full results): For positive affect, higher trait levels of creativity, social intelligence, and appreciation of beauty went along with a stronger positive association between states and positive affect (Figure 4, left panel). For negative affect, higher trait levels of creativity, social intelligence, and appreciation of beauty went along with a stronger negative association between states and negative affect (Figure 4, center panel). Further, trait hope and trait zest moderated the association between the associated states and negative affect, but in the opposite direction: Higher levels of trait hope and zest went along with a weaker connection between states and negative affect (Figure 4, right panel).

## 7 DISCUSSION

This study examined the daily enactments of character strengths and how they relate to character strengths traits and emotional well‐being. Using a daily diary design, we first aimed to apply whole trait theory to morally valued traits, as suggested previously (Jayawickreme et al., 2014; Jayawickreme & Fleeson, 2017). Our second aim was to test whether the previously reported between‐person relationships between character strengths and emotional well‐being can also be observed at the within‐person level.

First, our findings indicate that most character strength traits can generally be described as aggregates of character strengths states, in line with assumptions of whole trait theory. Trait scores of 18 of the 24 character strengths showed correlations of at least a large effect size. Overall, our results suggest that for most of the character strengths, the level of state–trait convergence is comparable to the levels of convergence reported for the personality traits of the five‐factor model (i.e., meta‐analytic correlations between 0.42 and 0.56; Fleeson & Gallagher, 2009).

However, the strength of the relationships between character strengths traits and aggregated states varied substantially between the 24 character strengths. We found that the emergence of character strengths (i.e., whether the character strength was rather tonic, i.e., less variable, or rather phasic, i.e., more variable) predicted the associations between traits and aggregated states: The character strengths with the lowest associations (bravery, teamwork, fairness, leadership, forgiveness, and self‐regulation) were—except fairness—all among the character strengths identified as most phasic (Arbenz et al., 2023). Displaying these character strengths may depend more strongly on situational characteristics, which may not have occurred regularly during the diary phase of 14 days, and we, therefore, observed a relatively low convergence between states and traits. Conversely, the character strengths with the highest trait–state convergence (spirituality, humor, gratitude, zest, hope, and social intelligence) tended to be perceived as rather tonic, that is, less variable (Arbenz et al., 2023). These character strengths seem less dependent on situational characteristics, which could be one of the reasons for their relatively strong trait–state convergence.

Second, we tested whether the amount of within‐person variability differed between the 24 character strengths. Again, we found that a character strength's tonic or phasic nature predicted the within‐person variability, which aligns with Peterson and Seligman's (2004) assumptions. For instance, the character strength of teamwork is considered rather phasic (i.e., more variable) and showed a stronger within‐person variation than the average character strength. Enacting teamwork is tied to certain situational characteristics that may not occur to the same extent on any given day, resulting in high day‐to‐day variability. It would be interesting to compare this pattern to studies assessing character strengths states with a higher resolution, that is, several times during the day, and with studies that either assess or control attributes of the situation.

Third, we investigated whether variability in character strength states would be related to character strength traits. We found that the character strength traits of perspective, honesty, social intelligence, and fairness related to a larger variability in character strengths states. None of the character strengths traits was related to a smaller variability in character strengths states. Wundrack et al. (2018) suggested on a theoretical basis that the ability to take different perspectives is furthered by variability in (personality) states, and the definitions of the character strengths of perspective, social intelligence, and fairness all pertain to perspective‐taking. Perspective is characterized by the ability to “provide wise counsel to others” and by “having ways of looking at the world that make sense to oneself and to other people” (Peterson & Seligman, 2004, p. 29) and Peterson and Seligman (2004, p. 29) describe social intelligence as “being aware of the motives and feelings of other people and oneself; knowing what to do to fit into different social situations; knowing what makes other people tick.” Further, perspective‐taking is a prerequisite for developing fairness‐associated moral reasoning (Peterson & Seligman, 2004). The character strength of honesty also contains the facet of authenticity, and higher authenticity has previously been linked with stronger within‐person variation in personality across different social roles (Sheldon et al., 1997). This notion also relates to the work on realness, described by Hopwood et al. (2021) as one aspect of authenticity, the tendency to act how one feels without considering direct consequences. Overall, these findings support the notions that variability in personality states may be considered a trait on its own (Baird et al., 2006; Fleeson, 2001; Noftle & Fleeson, 2015) and that this trait relates to the ability and tendency to consider others' perspectives (Wundrack et al., 2018). Further, the findings argue for the relevance of considering authenticity as a correlate of variability in personality states. More broadly, our findings may also be interpreted as supporting the idea that personality variability tends to be adaptive rather than maladaptive (Baird et al., 2006; Magee et al., 2018). To further examine this notion, it would be helpful also to consider contextual factors.

Fourth, addressing the second overarching aim of the present study, we investigated whether associations between character strengths and emotional well‐being at the between‐person level (Azañedo et al., 2014; Khumalo et al., 2008; Littman‐Ovadia & Lavy, 2012; Martínez‐Martí & Ruch, 2017) would extend to the within‐person level. We found that most character strengths that showed substantial between‐person relationships with positive affect (zest, hope, curiosity, perspective, social intelligence, creativity, gratitude, perseverance, and bravery) and with negative affect (hope, zest, self‐regulation, curiosity, perseverance, and humor) in previous studies also showed relationships in the same directions at the within‐person level in the present study. In contrast, the relationships of curiosity, self‐regulation, and humor with negative affect failed to replicate at the between‐person level (but all displayed the expected relationships at the within‐person level), while the associations with forgiveness failed to replicate at both the between‐ and within‐person level. Overall, our results largely align with previous studies on the associations between character strengths and emotional well‐being. Consequently, they support the implicit assumption underlying many character strengths‐based interventions that the between‐person relationships of character strengths and well‐being extend to within‐person relationships: Individuals indeed experience more positive and fewer negative emotions on days on which they show more creative or kind behavior, to name only two examples of character strengths.

However, there were also exceptions: Prudence showed an unexpected pattern of results. Prudence typically shows zero or small positive correlations with positive emotions at a dispositional level (e.g., Güsewell & Ruch, 2012; Wagner et al., 2020). Our results, however, showed a (slight) negative association with positive affect at the within‐person level and a positive association with negative affect at both the within‐ and between‐person level. That is, on days on which participants displayed more prudence than usual, they reported experiencing fewer positive and more negative affective states than usual, and participants who displayed prudence more frequently also tended to experience fewer positive affective states. While the effects found here are small and replication will be needed, two explanations seem plausible: First, thinking carefully before acting might, in some cases, prevent individuals from experiencing situations that could elicit positive affect. Second, adverse events that go along with negative affect might result in more prudent behavior.

In addition, the strengths of appreciation of beauty and excellence (ABE) and kindness showed considerably large associations with positive affect, both at the between‐ and within‐person level. While both strengths also showed meaningful associations with positive affect in previous studies (see Table 1), they are not typically considered among the “happiness strengths” (e.g., Littman‐Ovadia et al., 2017) and have even explicitly been considered as strengths with low correlations with life satisfaction in the context of an intervention study (Proyer et al., 2013). The findings regarding ABE and kindness suggest that defining “happiness strengths” solely based on between‐person effects might be shorthanded and should be complemented by findings on within‐person effects when deriving ideas for practical applications.

Several theoretical accounts have proposed that personality states partly explain the links between personality traits and well‐being (Howell et al., 2017; Kritzler et al., 2020; Wilt et al., 2012). Our results suggest that it would be worth studying this in greater detail regarding character strengths. In such efforts, it would also be relevant to include situational characteristics, as Kritzler et al. (2020) suggested, and examine to what extent character strengths states predict external criteria.

Fifth, we tested whether the trait level of a character strength moderates the relationships between character strength states and emotional well‐being. We found that this was not the case for most of the character strengths, in line with several previous studies that addressed this question for broader personality traits (e.g., Epley & Schroeder, 2014; Fleeson et al., 2002; Howell et al., 2017; Kritzler & Luhmann, 2021; Margolis & Lyubomirsky, 2020; van Allen et al., 2021). Only for the character strength of ABE, we found a pattern consistent with an effect of congruence between trait level and states: The relationship between state ABE and positive affect was stronger for those with high trait levels of ABE, that is, for those with high levels of ABE, displaying behavior in line with one's trait levels went along with higher positive affect. Further, contrary to the idea that trait–state congruence is related to higher well‐being, the negative link between state hope and state negative affect was stronger for individuals with lower levels of trait hope. Overall, our findings do not support the notion that an individual's trait levels are relevant to the relationship between enacting character strengths and emotional well‐being.

### 7.1 Implications

The present results may inform applications, namely character strengths‐based interventions (see Ruch et al., 2020; Schutte & Malouff, 2019). First, given that many interventions are based on encouraging the enactment of character strengths states, demonstrating the within‐person variability in character strengths states is crucial. Our results support the notion that substantial within‐person variability might be exploited to initiate changes (Gander et al., 2024). However, it is important to note that the present study observed naturally occurring fluctuations in character strength states, and these within‐person effects may not necessarily operate in the same manner as targeted changes, such as those implemented during interventions. Second, the findings suggest that the effectiveness of such interventions may not depend on an individual's trait level of a character strength. ABE might represent an exception, and future studies on the effectiveness of interventions focused on ABE (e.g., Martínez‐Martí et al., 2018; Proyer et al., 2016) should consider trait ABE as a moderator for effectiveness. Our results suggest that these interventions might be most effective for individuals high in ABE. In general, it might not be beneficial to focus specifically on the character strengths with high trait levels.

### 7.2 Limitations

Several limitations need to be considered in the interpretation of the present results. First, our sample consisted of German‐speaking adults who were predominantly female students. Although we did not observe any meaningful effects of age and gender in our analyses, the sample composition represents a relevant possible constraint on generality.

Second, we assessed character strength states by one item per character strength to limit participant burden. In contrast, character strengths traits were assessed using the VIA‐IS, containing 10 items per character strength. Thus, despite a relatively high convergence between the 1‐ and 10‐item measure (Ruch et al., 2014) and the one item containing a detailed description of the character strength, including several different behavioral manifestations, some of the variability between character strengths concerning the trait–state relationships might be explained by differences in the convergence between the trait and state items. Similarly, the present study used a 1‐item measure to assess states (not uncommon in diary studies, e.g., Conner & Silvia, 2015; Sherman et al., 2015), presumably limiting the associations with trait levels and positive and negative affect due to limited reliability.

Third, in creating the state assessment of character strengths (CSSRF), we adapted a trait questionnaire (CSRF) and used the original descriptions of the character strengths, which focused on stable between‐person descriptions to explain the concepts (e.g., “Creative people are…”) and asked participants how often they behaved according to this description today. While the current study demonstrated good convergence between these states and corresponding traits, it also revealed significant within‐person variability in states that systematically related to the emergence of character strengths, as well as positive and negative affect. However, future revisions of these items should place a greater emphasis on momentary experiences and activities, aligning with the recommended approaches for assessing personality states (Horstmann & Ziegler, 2020).

Fourth, we assessed character strength states daily, that is, at a relatively low resolution, to allow for sufficient opportunities to show the different character strengths across a day. However, a higher resolution of assessments might be required to detect more nuanced aspects of variability in character strengths, such as those caused by social roles (Bleidorn & Denissen, 2015), which might vary throughout the day.

Fifth, we did not consider differences between contexts and situations when investigating within‐person variability. For example, love of learning showed a relatively strong within‐person variability. Love of learning is enacted more frequently in education than in other life domains (Wagner et al., 2021). Since three‐quarters of the sample were university students, the variability may partly be due to a difference between the days they spent engaged in studying and the days they did not. Studies on personality states have pointed to the relevance of within‐ and across‐context variability (Beckmann et al., 2021; Geukes et al., 2017) and situation characteristics (e.g., Horstmann et al., 2021; Kritzler et al., 2020; Zachry et al., 2018), and this should be addressed as well in future studies on character strength states.

Moreover, our study was not designed to test more complex patterns of associations between states and traits beyond general convergence. For interests, it has been suggested that trait levels set the upper bounds for variability in associated states (Roemer et al., 2023), and similar complex patterns are conceivable for character strengths. Still, these will need to be explored in future studies designed specifically for this purpose. Finally, the present study did not use an experimental design, so we cannot make any assumptions about the directionality or causality of the effects, which should be considered when considering possible implications.

## 8 CONCLUSION

Can whole trait theory be applied to studying character strengths? Based on the present results, we argue in favor of “yes.” Overall, the relationships between aggregated character strength states and traits are comparable to those obtained for personality traits of the five‐factor model, showing that whole trait theory also applies to narrower, positively valued traits. The findings also highlight the role of the emergence of traits in understanding within‐person variability. Further, we demonstrate that character strengths and emotional well‐being are positively related when studying day‐to‐day experiences within individuals. We hope that future research will build on this work to enable a better understanding of the processes that link character strengths to well‐being and investigate changes in character strengths through the lens of character strength states.

## AUTHOR CONTRIBUTIONS

Lisa Wagner: Conceptualization, Investigation, Data Curation, Formal analysis, Writing—Original Draft, Writing—Review and Editing. Fabian Gander: Conceptualization, Investigation, Data Curation, Formal analysis, Writing—Original Draft, Writing—Review and Editing, Visualization.

## FUNDING INFORMATION

This study has been supported by a research grant from the Swiss National Science Foundation (100014_172723).

## CONFLICT OF INTEREST STATEMENT

We have no known conflict of interest to disclose.

## ETHICS STATEMENT

According to the local ethics committee guidelines, the study was exempt from ethical review.

## Data Availability

The supplementary tables, analysis scripts for the main analyses, and study materials are available at https://osf.io/mdtac/. The raw data can unfortunately not be made publicly available because the consent form used in this study explicitly excluded the disclosure of data to third parties.

## Endnotes

^1^
It should be noted that other, more complex patterns of associations between traits and states have also been proposed: For example, Roemer et al. (2023) reported that interest states vary below the corresponding trait level, that is, that interest states are unlikely to exceed the trait level. While this is an intriguing approach, it is beyond the scope of the present manuscript to also examining more specific patterns of character‐related trait–state associations.

^2^
When predicting kindness states by the intercept (RQ2), the models failed to converge due to five participants reporting the same levels of kindness across all measurement points. For these cases, we added a small random number (between −0.05 and 0.05) to the daily scores.

^3^
While in the other analyses character strengths are treated as fixed factors, in this analysis they are treated as random factors so that the general effect of trait level across all character strengths can be examined, but heterogeneity between character strengths is still considered. However, this means that the results of this analysis are not directly comparable to those of other analyses.

## References

[jopy12933-bib-0001] Arbenz, G. C. , Gander, F. , & Ruch, W. (2023). Breadth, polarity, and emergence of character strengths and their relevance for assessment. The Journal of Positive Psychology, 18(3), 383–393. 10.1080/17439760.2021.2018026

[jopy12933-bib-0002] Aust, F. , & Barth, M. (2022). papaja: Prepare reproducible APA journal articles with R Markdown . https://github.com/crsh/papaja

[jopy12933-bib-0003] Azañedo, C. M. , Fernández‐Abascal, E. G. , & Barraca, J. (2014). Character strengths in Spain: Validation of the Values in Action Inventory of Strengths (VIA‐IS) in a Spanish sample. Clínica y Salud, 25(2), 123–130. 10.1016/j.clysa.2014.06.002

[jopy12933-bib-0004] Baird, B. M. , Le, K. , & Lucas, R. E. (2006). On the nature of intraindividual personality variability: Reliability, validity, and associations with well‐being. Journal of Personality and Social Psychology, 90(3), 512–527. 10.1037/0022-3514.90.3.512 16594835

[jopy12933-bib-0005] Bates, D. , Mächler, M. , Bolker, B. , & Walker, S. (2015). Fitting linear mixed‐effects models using lme4. Journal of Statistical Software, 67(1), 1–48. 10.18637/jss.v067.i01

[jopy12933-bib-0006] Baumann, D. , Ruch, W. , Margelisch, K. , Gander, F. , & Wagner, L. (2020). Character strengths and life satisfaction in later life: An analysis of different living conditions. Applied Research in Quality of Life, 15(2), 329–347. 10.1007/s11482-018-9689-x

[jopy12933-bib-0007] Beckmann, N. , Birney, D. P. , Minbashian, A. , & Beckmann, J. F. (2021). Personality dynamics at work: The effects of form, time, and context of variability. European Journal of Personality, 35(4), 421–449. 10.1177/08902070211017341

[jopy12933-bib-0008] Bleidorn, W. , & Denissen, J. J. A. (2015). Virtues in action—The new look of character traits. British Journal of Psychology, 106(4), 700–723. 10.1111/bjop.12117 25641361

[jopy12933-bib-0009] Bolger, N. , Davis, A. , & Rafaeli, E. (2003). Diary methods: Capturing life as it is lived. Annual Review of Psychology, 54(1), 579–616. 10.1146/annurev.psych.54.101601.145030 12499517

[jopy12933-bib-0010] Breil, S. M. , Schweppe, P. C. , Geukes, K. , Biesanz, J. C. , Quintus, M. , Wagner, J. , Wrzus, C. , Nestler, S. , & Back, M. D. (2022). The incremental validity of average states: A replication and extension of Finnigan and Vazire (2018). Journal of Personality and Social Psychology, 123(3), e23–e37. 10.1037/pspp0000408 35113627

[jopy12933-bib-0011] Bruna, M. O. , Brabete, A. C. , & Izquierdo, J. M. A. (2019). Reliability generalization as a seal of quality of substantive meta‐analyses: The case of the VIA Inventory of Strengths (VIA‐IS) and their relationships to life satisfaction. Psychological Reports, 122(3), 1167–1188. 10.1177/0033294118779198 29848214

[jopy12933-bib-0012] Bürkner, P.‐C. (2021). Bayesian item response modeling in R with brms and Stan. Journal of Statistical Software, 100(5), 1–54. 10.18637/jss.v100.i05

[jopy12933-bib-0013] Buschor, C. , Proyer, R. T. , & Ruch, W. (2013). Self‐ and peer‐rated character strengths: How do they relate to satisfaction with life and orientations to happiness? The Journal of Positive Psychology, 8(2), 116–127. 10.1080/17439760.2012.758305

[jopy12933-bib-0014] Conner, T. S. , & Silvia, P. J. (2015). Creative days: A daily diary study of emotion, personality, and everyday creativity. Psychology of Aesthetics, Creativity, and the Arts, 9(4), 463–470. 10.1037/aca0000022.supp

[jopy12933-bib-0015] Curran, P. J. , & Bauer, D. J. (2011). The disaggregation of within‐person and between‐person effects in longitudinal models of change. Annual Review of Psychology, 62(1), 583–619. 10.1146/annurev.psych.093008.100356 PMC3059070 19575624

[jopy12933-bib-0016] Dahlke, J. A. , & Wiernik, B. M. (2019). psychmeta: An R package for psychometric meta‐analysis. Applied Psychological Measurement, 43(5), 415–416. 10.1177/0146621618795933 31235986 PMC6572911

[jopy12933-bib-0017] Denissen, J. J. A. , Luhmann, M. , Chung, J. M. , & Bleidorn, W. (2019). Transactions between life events and personality traits across the adult lifespan. Journal of Personality and Social Psychology, 116(4), 612–633. 10.1037/pspp0000196 30047764

[jopy12933-bib-0018] Diener, E. , Emmons, R. A. , Larsen, R. J. , & Griffin, S. (1985). The satisfaction with life scale. Journal of Personality Assessment, 49(1), 71–75. 10.1207/s15327752jpa4901_13 16367493

[jopy12933-bib-0019] Epley, N. , & Schroeder, J. (2014). Mistakenly seeking solitude. Journal of Experimental Psychology: General, 143(5), 1980–1999. 10.1037/a0037323 25019381

[jopy12933-bib-0020] Finnigan, K. M. , & Vazire, S. (2018). The incremental validity of average state self‐reports over global self‐reports of personality. Journal of Personality and Social Psychology, 115(2), 321–337. 10.1037/pspp0000136 28277717

[jopy12933-bib-0021] Fleeson, W. (2001). Toward a structure‐ and process‐integrated view of personality: Traits as density distributions of states. Journal of Personality and Social Psychology, 80(6), 1011–1027. 10.1037/0022-3514.80.6.1011 11414368

[jopy12933-bib-0022] Fleeson, W. , Furr, R. M. , Jayawickreme, E. , Helzer, E. G. , Hartley, A. G. , & Meindl, P. (2015). Personality science and the foundations of character. In C. B. Miller , R. M. Furr , A. Knobel , & W. Fleeson (Eds.), Character: New directions from philosophy, psychology, and theology (pp. 41–71). Oxford University Press.

[jopy12933-bib-0023] Fleeson, W. , & Gallagher, P. (2009). The implications of Big Five standing for the distribution of trait manifestation in behavior: Fifteen experience‐sampling studies and a meta‐analysis. Journal of Personality and Social Psychology, 97(6), 1097–1114. 10.1037/a0016786 19968421 PMC2791901

[jopy12933-bib-0024] Fleeson, W. , & Jayawickreme, E. (2015). Whole trait theory. Journal of Research in Personality, 56, 82–92. 10.1016/j.jrp.2014.10.009 26097268 PMC4472377

[jopy12933-bib-0025] Fleeson, W. , & Leicht, C. (2006). On delineating and integrating the study of variability and stability in personality psychology: Interpersonal trust as illustration. Journal of Research in Personality, 40(1), 5–20. 10.1016/j.jrp.2005.08.004

[jopy12933-bib-0026] Fleeson, W. , Malanos, A. B. , & Achille, N. M. (2002). An intraindividual process approach to the relationship between extraversion and positive affect: Is acting extraverted as “good” as being extraverted? Journal of Personality and Social Psychology, 83(6), 1409–1422. 10.1037/0022-3514.83.6.1409 12500821

[jopy12933-bib-0027] Gallagher, P. , Fleeson, W. , & Hoyle, R. H. (2011). A self‐regulatory mechanism for personality trait stability: Contra‐trait effort. Social Psychological and Personality Science, 2(4), 335–342. 10.1177/1948550610390701

[jopy12933-bib-0028] Gander, F. , Hofmann, J. , Proyer, R. T. , & Ruch, W. (2020). Character strengths—Stability, change, and relationships with well‐being changes. Applied Research in Quality of Life, 15(2), 349–367. 10.1007/s11482-018-9690-4 PMC7250648 32457813

[jopy12933-bib-0029] Gander, F. , Wagner, L. , Amann, L. , & Ruch, W. (2022). What are character strengths good for? A daily diary study on character strengths enactment. The Journal of Positive Psychology, 17(5), 718–728. 10.1080/17439760.2021.1926532

[jopy12933-bib-0104] Gander, F. , Wagner, L. , & Niemiec, R. M. (2024). Do character strengths‐based interventions change character strengths? Two randomized controlled intervention studies. Collabra. Psychology, 10(1). 10.1525/collabra.108604

[jopy12933-bib-0030] Geukes, K. , Nestler, S. , Hutteman, R. , Küfner, A. C. P. , & Back, M. D. (2017). Trait personality and state variability: Predicting individual differences in within‐ and cross‐context fluctuations in affect, self‐evaluations, and behavior in everyday life. Journal of Research in Personality, 69, 124–138. 10.1016/j.jrp.2016.06.003

[jopy12933-bib-0031] Gignac, G. E. , & Szodorai, E. T. (2016). Effect size guidelines for individual differences researchers. Personality and Individual Differences, 102, 74–78. 10.1016/j.paid.2016.06.069

[jopy12933-bib-0032] Green, P. , & MacLeod, C. J. (2016). SIMR: An R package for power analysis of generalized linear mixed models by simulation. Methods in Ecology and Evolution, 7(4), 493–498. 10.1111/2041-210X.12504

[jopy12933-bib-0033] Grolemund, G. , & Wickham, H. (2011). Dates and times made easy with lubridate. Journal of Statistical Software, 40(3), 1–25. 10.18637/jss.v040.i03

[jopy12933-bib-0034] Güsewell, A. , & Ruch, W. (2012). Are only emotional strengths emotional? Character strengths and disposition to positive emotions. Applied Psychology: Health and Well‐Being, 4(2), 218–239. 10.1111/j.1758-0854.2012.01070.x 26286979

[jopy12933-bib-0035] Hamaker, E. L. (2012). Why researchers should think “within‐person”: A paradigmatic rationale. In M. R. Mehl , & T. S. Conner (Eds.), Handbook of research methods for studying daily life (pp. 43–61). The Guilford Press.

[jopy12933-bib-0036] Hartshorne, H. , & May, M. A. (1928). Studies in the nature of character: Studies in deceit. MacMillan Co. 10.1037/13386-000

[jopy12933-bib-0037] Heisig, J. P. , & Schaeffer, M. (2019). Why you should always include a random slope for the lower‐level variable involved in a cross‐level interaction. European Sociological Review, 35(2), 258–279. 10.1093/esr/jcy053

[jopy12933-bib-0038] Hopwood, C. J. , Good, E. W. , Levendosky, A. A. , Zimmermann, J. , Dumat, D. , Finkel, E. J. , Eastwick, P. E. , & Bleidorn, W. (2021). Realness is a core feature of authenticity. Journal of Research in Personality, 92, 104086. 10.1016/j.jrp.2021.104086

[jopy12933-bib-0039] Horstmann, K. T. , Rauthmann, J. F. , Sherman, R. A. , & Ziegler, M. (2021). Unveiling an exclusive link: Predicting behavior with personality, situation perception, and affect in a preregistered experience sampling study. Journal of Personality and Social Psychology, 120(5), 1317–1343. 10.1037/pspp0000357 32940516

[jopy12933-bib-0040] Horstmann, K. T. , & Ziegler, M. (2020). Assessing personality states: What to consider when constructing personality state measures. European Journal of Personality, 34(6), 1037–1059. 10.1002/per.2266

[jopy12933-bib-0041] Howell, R. T. , Ksendzova, M. , Nestingen, E. , Yerahian, C. , & Iyer, R. (2017). Your personality on a good day: How trait and state personality predict daily well‐being. Journal of Research in Personality, 69, 250–263. 10.1016/j.jrp.2016.08.001

[jopy12933-bib-0042] Jacques‐Hamilton, R. , Sun, J. , & Smillie, L. D. (2019). Costs and benefits of acting extraverted: A randomized controlled trial. Journal of Experimental Psychology: General, 148(9), 1538–1556. 10.1037/xge0000516 30489119

[jopy12933-bib-0043] Jayawickreme, E. , & Fleeson, W. (2017). Does whole trait theory work for the virtues? In W. Sinnott‐Armstrong & C. B. Miller (Eds.), Moral psychology (pp. 75–103). The MIT Press. 10.7551/mitpress/9245.003.0005

[jopy12933-bib-0044] Jayawickreme, E. , Meindl, P. , Helzer, E. G. , Furr, R. M. , & Fleeson, W. (2014). Virtuous states and virtuous traits: How the empirical evidence regarding the existence of broad traits saves virtue ethics from the situationist critique. Theory and Research in Education, 12(3), 283–308. 10.1177/1477878514545206

[jopy12933-bib-0045] Jayawickreme, E. , Zachry, C. E. , & Fleeson, W. (2019). Whole trait theory: An integrative approach to examining personality structure and process. Personality and Individual Differences, 136, 2–11. 10.1016/j.paid.2018.06.045

[jopy12933-bib-0046] Judge, T. A. , Simon, L. S. , Hurst, C. , & Kelley, K. (2014). What I experienced yesterday is who I am today: Relationship of work motivations and behaviors to within‐individual variation in the five‐factor model of personality. Journal of Applied Psychology, 99(2), 199–221. 10.1037/a0034485 24099348

[jopy12933-bib-0047] Kammann, R. , & Flett, R. (1983). Affectometer 2: A scale to measure current level of general happiness. Australian Journal of Psychology, 35(2), 259–265. 10.1080/00049538308255070

[jopy12933-bib-0048] Kelley, K. (2022). MBESS: The MBESS r package . https://CRAN.R‐project.org/package=MBESS

[jopy12933-bib-0049] Khumalo, I. P. , Wissing, M. P. , & Temane, Q. M. (2008). Exploring the validity of the Values‐In‐Action Inventory of Strengths (VIA‐IS) in an African context. Journal of Psychology in Africa, 18(1), 133–142. 10.1080/14330237.2008.10820180

[jopy12933-bib-0050] Kritzler, S. , Krasko, J. , & Luhmann, M. (2020). Inside the happy personality: Personality states, situation experience, and state affect mediate the relation between personality and affect. Journal of Research in Personality, 85, 103929. 10.1016/j.jrp.2020.103929

[jopy12933-bib-0051] Kritzler, S. , & Luhmann, M. (2021). Be yourself and behave appropriately: Exploring associations between incongruent personality states and positive affect, tiredness, and cognitive performance. Collabra: Psychology, 7(1), 27386. 10.1525/collabra.27386

[jopy12933-bib-0052] Latané, B. , & Rodin, J. (1969). A lady in distress: Inhibiting effects of friends and strangers on bystander intervention. Journal of Experimental Social Psychology, 5(2), 189–202. 10.1016/0022-1031(69)90046-8

[jopy12933-bib-0053] Littman‐Ovadia, H. , & Lavy, S. (2012). Character strengths in Israel: Hebrew adaptation of the VIA inventory of strengths. European Journal of Psychological Assessment, 28(1), 41–50. 10.1027/1015-5759/a000089

[jopy12933-bib-0054] Littman‐Ovadia, H. , Lavy, S. , & Boiman‐Meshita, M. (2017). When theory and research collide: Examining correlates of signature strengths use at work. Journal of Happiness Studies, 18(2), 527–548. 10.1007/s10902-016-9739-8

[jopy12933-bib-0055] Long, J. A. (2021). *Interactions: Comprehensive, user‐friendly toolkit for probing interactions* (1.1.5). https://interactions.jacob‐long.com/

[jopy12933-bib-0056] Lüdecke, D. (2023). sjPlot: Data visualization for statistics in social science . https://CRAN.R‐project.org/package=sjPlot

[jopy12933-bib-0057] Lüdecke, D. , Ben‐Shachar, M. S. , Patil, I. , & Makowski, D. (2020). Extracting, computing and exploring the parameters of statistical models using R. Journal of Open Source Software, 5(53), 2445. 10.21105/joss.02445

[jopy12933-bib-0058] Mader, N. , Arslan, R. C. , Schmukle, S. C. , & Rohrer, J. M. (2023). Emotional (in)stability: Neuroticism is associated with increased variability in negative emotion after all. Proceedings of the National Academy of Sciences of the United States of America, 120(23), e2212154120. 10.1073/pnas.2212154120 37253012 PMC10266024

[jopy12933-bib-0059] Magee, C. , Buchtel, E. E. , Human, L. J. , Murray, D. R. , & Biesanz, J. C. (2018). Is personality variability associated with adjustment? Journal of Research in Personality, 72, 22–43. 10.1016/j.jrp.2016.08.005

[jopy12933-bib-0060] Makowski, D. , Ben‐Shachar, M. S. , Patil, I. , & Lüdecke, D. (2020). Methods and algorithms for correlation analysis in R. Journal of Open Source Software, 5(51), 2306. 10.21105/joss.02306

[jopy12933-bib-0061] Margolis, S. , & Lyubomirsky, S. (2020). Experimental manipulation of extraverted and introverted behavior and its effects on well‐being. Journal of Experimental Psychology: General, 149(4), 719–731. 10.1037/xge0000668 31368759

[jopy12933-bib-0062] Martínez‐Martí, M. L. , Avia, M. D. , & Hernández‐Lloreda, M. J. (2018). Effects of an appreciation of beauty randomized‐controlled trial web‐based intervention on appreciation of beauty and well‐being. Psychology of Aesthetics, Creativity, and the Arts, 12(3), 272–283. 10.1037/aca0000164

[jopy12933-bib-0063] Martínez‐Martí, M. L. , & Ruch, W. (2017). Character strengths predict resilience over and above positive affect, self‐efficacy, optimism, social support, self‐esteem, and life satisfaction. The Journal of Positive Psychology, 12(2), 110–119. 10.1080/17439760.2016.1163403

[jopy12933-bib-0064] McGrath, R. E. (2014). Scale‐ and item‐level factor analyses of the VIA inventory of strengths. Assessment, 21(1), 4–14. 10.1177/1073191112450612 22855509

[jopy12933-bib-0065] McGrath, R. E. , Hall‐Simmonds, A. , & Goldberg, L. R. (2020). Are measures of character and personality distinct? Evidence from observed‐score and true‐score analyses. Assessment, 27(1), 117–135. 10.1177/1073191117738047 29073771 PMC5878981

[jopy12933-bib-0066] Meindl, P. , Jayawickreme, E. , Furr, R. M. , & Fleeson, W. (2015). A foundation beam for studying morality from a personological point of view: Are individual differences in moral behaviors and thoughts consistent? Journal of Research in Personality, 59, 81–92. 10.1016/j.jrp.2015.09.005

[jopy12933-bib-0067] Moskowitz, D. S. , & Coté, S. (1995). Do interpersonal traits predict affect? A comparison of three models. Journal of Personality and Social Psychology, 69(5), 915–924. 10.1037/0022-3514.69.5.915

[jopy12933-bib-0068] Mroczek, D. K. , & Kolarz, C. M. (1998). The effect of age on positive and negative affect: A developmental perspective on happiness. Journal of Personality and Social Psychology, 75(5), 1333–1349. 10.1037/0022-3514.75.5.1333 9866191

[jopy12933-bib-0069] Noftle, E. E. , & Fleeson, W. (2015). Intraindividual variability in adult personality development. In M. Diehl , K. Hooker , & M. J. Sliwinski (Eds.), Handbook of intraindividual variability across the life span (pp. 176–197). Routledge/Taylor & Francis Group.

[jopy12933-bib-0070] Nübold, A. , & Hülsheger, U. R. (2021). Personality states mediate the effect of a mindfulness intervention on employees' work outcomes: A randomized controlled trial. European Journal of Personality, 35(4), 646–664. 10.1177/08902070211012915

[jopy12933-bib-0071] Park, N. , & Peterson, C. (2007). Methodological issues in positive psychology and the assessment of character strengths. In A. D. Ong , & M. H. M. van Dulmen (Eds.),Oxford handbook of methods in positive psychology (pp. 292–305). Oxford University Press.

[jopy12933-bib-0072] Park, N. , Peterson, C. , & Seligman, M. E. P. (2004). Strengths of character and well‐being. Journal of Social and Clinical Psychology, 23(5), 603–619. 10.1521/jscp.23.5.603.50748

[jopy12933-bib-0073] Peterson, C. , & Seligman, M. E. P. (2004). Character strengths and virtues: A handbook and classification. American Psychological Association; Oxford University Press.

[jopy12933-bib-0074] Prentice, M. , Jayawickreme, E. , & Fleeson, W. (2020). An experience sampling study of the momentary dynamics of moral, autonomous, competent, and related need satisfactions, moral enactments, and psychological thriving. Motivation and Emotion, 44(2), 244–256. 10.1007/s11031-020-09829-3

[jopy12933-bib-0075] Proyer, R. T. , Gander, F. , Wellenzohn, S. , & Ruch, W. (2016). Nine beautiful things: A self‐administered online positive psychology intervention on the beauty in nature, arts, and behaviors increases happiness and ameliorates depressive symptoms. Personality and Individual Differences, 94, 189–193. 10.1016/j.paid.2016.01.028

[jopy12933-bib-0076] Proyer, R. T. , Gander, F. , Wyss, T. , & Ruch, W. (2011). The relation of character strengths to past, present, and future life satisfaction among German‐speaking women. Applied Psychology: Health and Well‐Being, 3(3), 370–384. 10.1111/j.1758-0854.2011.01060.x

[jopy12933-bib-0077] Proyer, R. T. , Ruch, W. , & Buschor, C. (2013). Testing strengths‐based interventions: A preliminary study on the effectiveness of a program targeting curiosity, gratitude, hope, humor, and zest for enhancing life satisfaction. Journal of Happiness Studies, 14(1), 275–292. 10.1007/s10902-012-9331-9

[jopy12933-bib-0078] R Core Team . (2023). R: A language and environment for statistical computing. R Foundation for Statistical Computing. https://www.R‐project.org/

[jopy12933-bib-0079] Rauthmann, J. F. , Horstmann, K. T. , & Sherman, R. A. (2019). Do self‐reported traits and aggregated states capture the same thing? A nomological perspective on trait‐state homomorphy. Social Psychological and Personality Science, 10(5), 596–611. 10.1177/1948550618774772

[jopy12933-bib-0080] Revelle, R. (2023). Psych: Procedures for psychological, psychometric, and personality research. Northwestern University. https://CRAN.R‐project.org/package=psych

[jopy12933-bib-0081] Ringwald, W. R. , Manuck, S. B. , Marsland, A. L. , & Wright, A. G. C. (2022). Psychometric evaluation of a big five personality state scale for intensive longitudinal studies. Assessment, 29(6), 1301–1319. 10.1177/1073191121100825 33949209 PMC9832333

[jopy12933-bib-0082] Roemer, L. , Stoll, G. , Rounds, J. , & Ziegler, M. (2023). Why does the trait‐state relation in vocational interests differ from that in personality? Exploring interest variability in daily life. Journal of Research in Personality, 105, 104386. 10.1016/j.jrp.2023.104386

[jopy12933-bib-0083] Ruch, W. , Martínez‐Martí, M. L. , Proyer, R. T. , & Harzer, C. (2014). The character strengths rating form (CSRF): Development and initial assessment of a 24‐item rating scale to assess character strengths. Personality and Individual Differences, 68, 53–58. 10.1016/j.paid.2014.03.042

[jopy12933-bib-0084] Ruch, W. , Niemiec, R. M. , McGrath, R. E. , Gander, F. , & Proyer, R. T. (2020). Character strengths‐based interventions: Open questions and ideas for future research. The Journal of Positive Psychology, 15(5), 680–684. 10.1080/17439760.2020.1789700

[jopy12933-bib-0085] Ruch, W. , Proyer, R. T. , Harzer, C. , Park, N. , Peterson, C. , & Seligman, M. E. P. (2010). Values in action inventory of strengths (VIA‐IS): Adaptation and validation of the German version and the development of a peer‐rating form. Journal of Individual Differences, 31(3), 138–149. 10.1027/1614-0001/a000022

[jopy12933-bib-0086] Ruch, W. , Vylobkova, V. , & Heintz, S. (2023). Two of a kind or distant relatives? A multimethod in vestigation of the overlap between personality traits and character strengths. Journal of Individual Differences, 44(4), 263–270. 10.1027/1614-0001/a000400

[jopy12933-bib-0087] Scheinin, I. , Kalimeri, M. , Jagerroos, V. , Parkkinen, J. , Tikkanen, E. , Würtz, P. , & Kangas, A. (2023). ggforestplot: Forestplots of measures of effects and their confidence intervals . https://nightingalehealth.github.io/ggforestplot/index.html

[jopy12933-bib-0088] Schutte, N. S. , & Malouff, J. M. (2019). The impact of signature character strengths interventions: A meta‐analysis. Journal of Happiness Studies, 20(4), 1179–1196. 10.1007/s10902-018-9990-2

[jopy12933-bib-0089] Sheldon, K. M. , Ryan, R. M. , Rawsthorne, L. J. , & Ilardi, B. (1997). Trait self and true self: Cross‐role variation in the Big‐Five personality traits and its relations with psychological authenticity and subjective well‐being. Journal of Personality and Social Psychology, 73(6), 1380–1393. 10.1037/0022-3514.73.6.1380

[jopy12933-bib-0090] Sherman, R. A. , Rauthmann, J. F. , Brown, N. A. , Serfass, D. G. , & Jones, A. B. (2015). The independent effects of personality and situations on real‐time expressions of behavior and emotion. Journal of Personality and Social Psychology, 109(5), 872–888. 10.1037/pspp0000036 25915131

[jopy12933-bib-0091] Signorell, A. (2023). DescTools: Tools for descriptive statistics . https://CRAN.R‐project.org/package=DescTools

[jopy12933-bib-0092] Stahlmann, A. G. , & Ruch, W. (2020). Scrutinizing the criteria for character strengths: Laypersons assert that every strength is positively morally valued, even in the absence of tangible outcomes. Frontiers in Psychology, 11, 591028. 10.3389/fpsyg.2020.591028 33101158 PMC7554639

[jopy12933-bib-0093] van Allen, Z. M. , Walker, D. L. , Streiner, T. , & Zelenski, J. M. (2021). Enacted extraversion as a well‐being enhancing strategy in everyday life: Testing across three, week‐long interventions. Collabra: Psychology, 7(1), 29931. 10.1525/collabra.29931

[jopy12933-bib-0094] Wagner, L. , Gander, F. , Proyer, R. T. , & Ruch, W. (2020). Character strengths and PERMA: Investigating the relationships of character strengths with a multidimensional framework of well‐being. Applied Research in Quality of Life, 15(2), 307–328. 10.1007/s11482-018-9695-z

[jopy12933-bib-0095] Wagner, L. , Pindeus, L. , & Ruch, W. (2021). Character strengths in the life domains of work, education, leisure, and relationships, and their associations with flourishing. Frontiers in Psychology, 12, 597534. 10.3389/fpsyg.2021.597534 33967881 PMC8096931

[jopy12933-bib-0096] Wagner, L. , & Ruch, W. (2023). Displaying character strengths in behavior is related to well‐being and achievement at school: Evidence from between‐ and within‐person analyses. The Journal of Positive Psychology, 18(3), 460–480. 10.1080/17439760.2022.2109196

[jopy12933-bib-0097] Watson, D. , Clark, L. A. , & Tellegen, A. (1988). Development and validation of brief measures of positive and negative affect: The PANAS scales. Journal of Personality and Social Psychology, 54(6), 1063–1070. 10.1037/0022-3514.54.6.1063 3397865

[jopy12933-bib-0098] Wickham, H. , Averick, M. , Bryan, J. , Chang, W. , McGowan, L. D. , François, R. , Grolemund, G. , Hayes, A. , Henry, L. , Hester, J. , Kuhn, M. , Pedersen, T. L. , Miller, E. , Bache, S. M. , Müller, K. , Ooms, J. , Robinson, D. , Seidel, D. P. , Spinu, V. , … Yutani, H. (2019). Welcome to the Tidyverse. Journal of Open Source Software, 4(43), 1686. 10.21105/joss.01686

[jopy12933-bib-0099] Williams, D. R. , Martin, S. R. , Liu, S. , & Rast, P. (2020). Bayesian multivariate mixed‐effects location scale modeling of longitudinal relations among affective traits, states, and physical activity. European Journal of Psychological Assessment, 36(6), 981–997. 10.1027/1015-5759/a000624 34764628 PMC8580300

[jopy12933-bib-0100] Wilt, J. , Noftle, E. E. , Fleeson, W. , & Spain, J. S. (2012). The dynamic role of personality states in mediating the relationship between extraversion and positive affect. Journal of Personality, 80(5), 1205–1236. 10.1111/j.1467-6494.2011.00756.x 22092066 PMC3492883

[jopy12933-bib-0101] Wundrack, R. , Prager, J. , Asselmann, E. , O'Connell, G. , & Specht, J. (2018). Does intraindividual variability of personality states improve perspective taking? An ecological approach integrating personality and social cognition. Journal of Intelligence, 6(4), 50. 10.3390/jintelligence6040050 31162477 PMC6480758

[jopy12933-bib-0102] Zachry, C. E. , Phan, L. V. , Blackie, L. E. R. , & Jayawickreme, E. (2018). Situation‐based contingencies underlying wisdom‐content manifestations: Examining intellectual humility in daily life. The Journals of Gerontology: Series B, 73(8), 1404–1415. 10.1093/geronb/gby016 29474696

[jopy12933-bib-0103] Zuroff, D. C. , Sadikaj, G. , Kelly, A. C. , & Leybman, M. J. (2016). Conceptualizing and measuring self‐criticism as both a personality trait and a personality state. Journal of Personality Assessment, 98(1), 14–21. 10.1080/00223891.2015.1044604 26046620

